# Development of Chinese genetic reference panel for Fragile X Syndrome and its application to the screen of 10,000 Chinese pregnant women and women planning pregnancy

**DOI:** 10.1002/mgg3.1236

**Published:** 2020-04-12

**Authors:** Fei Gao, Wen Huang, Yanjun You, Jie Huang, Juan Zhao, Jin Xue, Huaixing Kang, Yingbao Zhu, Zhengmao Hu, Emily G. Allen, Peng Jin, Kun Xia, Ranhui Duan

**Affiliations:** ^1^ Center for Medical Genetics School of Life Sciences Central South University Changsha Hunan China; ^2^ National Institutes for Food and Drug Control Beijing China; ^3^ Hunan Key Laboratory of Medical Genetics Central South University Changsha Hunan China; ^4^ Department of Human Genetics Emory University School of Medicine Atlanta GA USA; ^5^ Hunan Key Laboratory of Animal Models for Human Diseases Central South University Changsha Hunan China

**Keywords:** CGG repeat, *FMR1*, Fragile X Syndrome

## Abstract

**Background:**

Fragile X syndrome (FXS) is the most common inherited form of intellectual disability caused by a CGG repeat expansion in the 5′ untranslated region of the *FMR1* gene. When the number of repeats exceeds 200, the gene becomes hypermethylated and is transcriptionally silenced, resulting in FXS. Other allelic forms of the gene that are studied because of their instability or phenotypic consequence include intermediate alleles (45–54 CGG repeats) and premutation alleles (55–200 repeats). Normal alleles are classified as having <45 CGG repeats. Population screening studies have been conducted among American and Australian populations; however, large population‐based studies have not been completed in China.

**Methods and Results:**

In this work we present FXS screening results from 10,145 women of childbearing age from China. We first created and tested a standard panel that was comprised of normal, intermediate, premutation, and full mutation samples, and we performed the screening after confirming the consistency of genotyping results among laboratories.

**Conclusion:**

Based on our findings, we have determined the intermediate and premutation carrier prevalence of 1/130 and 1/634, respectively, among Chinese women.

## Graphical abstract

FXS is the most common inherited form of intellectual disability. Population screening studies have been conducted among American and Australian populations; however, large population‐based studies have not been completed in China. In this work we present FXS screening results from more than 10,000 women of child bearing age from China. We first created and tested a standard panel that was comprised of normal, intermediate, premutation, and full mutation samples. We have determined the intermediate carrier frequency as 1/130 among Chinese women, and the premutation carrier frequency as 1/634 women among Chinese women.

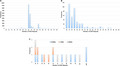

## 1 INTRODUCTION

Fragile X Syndrome (FXS, OMIM300624) is the most common monogenic disease that causes hereditary mental retardation and autism spectrum disorders. In approximately 99% of FXS patients, the loss of fragile X mental retardation protein expression is caused by an expansion of a CGG repeat within the 5′ untranslated region of the fragile X mental retardation 1 (*FMR1,* OMIM309550) gene (Kelleher & Bear, 2008). The number of CGG repeats is highly polymorphic in the population. Individuals with less than 45 repeats fall into the normal range, 45–54 repeats fall into the intermediate or grey zone, those with 55–200 repeats are premutation carriers, and individuals with over 200 repeats have the full mutation.

The frequency of fragile X carriers is high, and different phenotypes and pathogenic mechanisms are seen in premutations in addition to the phenotypes seen in full mutation individuals, indicating a need for fragile X screening. The incidence of FXS differs for racial and ethnic groups. It has been reported that the prevalence of males with FXS range from 1/3,717 to 1/8,918 in the European and American population (Crawford et al., 2002; Jacobs et al., 1993), while the rate is 1/2,545 in African Americans (Crawford et al., 2002). The fragile X premutation frequency in females is very high: estimates have reached 1/130 to 1/260 in European and American populations (Hantash et al., 2011; Hunter et al., 2014; Maenner et al., 2013; Seltzer et al., 2012; Yrigollen et al., 2012). However, the rate is ~ 1/400 in Chinese American population (Owens et al., 2018). Approximately one third of premutation males and a small number of females will develop Fragile X–associated tremor/ataxia syndrome after age 50 (Berry‐Kravis et al., 2007; Hagerman & Hagerman, 2016), and approximately 20% of female premutation carriers will develop fragile X–associated primary ovarian insufficiency (Allen et al., 2007; Sherman, 2000). Full mutation alleles have more than 200 CGG repeats, and are hypermethylated and transcriptionally silenced (Fu et al., 1991; Verkerk et al., 1991). All males with a full mutation will develop FXS, and about half of females with the full mutations will develop FXS due to the X‐linked nature of the gene. A female premutation carrier may have no phenotype at the time of marriage and childbearing, and, in many cases, only after the transmission of an unstable CGG expansion resulting in a child with FXS, the mutation is identified. Therefore, screening all pregnant women for fragile X carrier status can help female carriers to understand risks and choices for family and reproductive planning. Recently, the results on newborn screening pilot studies on newborns and pregnant women for fragile X carrier status in the United States and Australia have been published (Archibald et al., 2018; Bailey et al., 2017; Hantash et al., 2011; Yrigollen et al., 2012). However, the molecular techniques for determining *FMR1* repeat size are challenging, and the current availability of genetic counseling cannot satisfy the demand of pregnant women, leading to concerns on widespread availability of newborn screening (Bailey et al., 2017; Finucane et al., 2012; Finucane, Lincoln, Bailey, & Martin, 2017). Thus, methods to most effectively promote FXS screening have become a focus in the prenatal screening field.

In China, a unified system for screening of FXS in women of childbearing age has not been established. Although most women are aware of Down Syndrome, few have an understanding of FXS. Some medical professionals have knowledge of FXS, however, the majority do not have firsthand experience with FXS families (Li, Huang, Luo, Lin, & Duan, 2013). Therefore, they may not be fully aware of the pathogenesis and of the complicated genetics of FXS. In addition to the cognitive phenotypes associated with FXS, challenges with FXS detection technology also limit its clinical application in China. As CGG repeats increase in size, alleles are more difficult for PCR amplification, resulting in a sharp decrease in efficiency of DNA polymerase or even lack of amplification. FXS classification is complicated: screening for FXS not only has to accurately distinguish the intermediate‐zone, premutation, and full mutation alleles but also needs to identify the premutation/full mutation size mosaicism and methylation/nonmethylation mosaicism. Although commercial screening kits for *FMR1* CGG repeat PCR and *FMR1* methylation status which may resolve the above problems are available in China, the high cost of the equipment and kits, the complicated results of the test, and the need for skilled inspectors restrict their wide application. Noninvasive prenatal screening is highly accepted in China, and there is an increasing appeal for fragile X carrier screening. Thresholds for normal, intermediate, premutation, and full mutation must be accurately determined, but there are currently no uniform reference standards for laboratories and commercial kits in China.

In 2008, the U.S. Center for Disease Control and Prevention and the Association for Molecular Pathology validated 16 cell lines with different CGG repeat sizes in 9 laboratories; however, increased variation was seen in alleles over 100 CGG repeats (Amos Wilson et al., 2008). In 2011, the World Health Organization launched a panel of FXS reference materials, and 21 laboratories from 17 countries participated in the verification. However, only one laboratory in Asia was involved in the testing of the five standard products that were generated. In addition, the sizing of larger alleles only defines samples as premutation range, not the exact size (Hawkins et al., 2011). Purchasing of FXS standards is difficult in China. In order to promote the fragile X carrier screening, the China Food and Drug Testing Institute collaborated with five laboratories, and performed the comparison and verification of normal, intermediate, premutation and full mutation alleles from 17 DNA samples. Normal, intermediate, and premutation sample results from different laboratories were compared, and Southern blot was also used for further validation. Ultimately, the reference sizes were accurately determined illustrating the ability of all laboratories to establish consistent results across FXS detection platforms.

The frequency of female fragile X carriers is relatively high; however, prevalence varies based on ethnic differences (Crawford, Acuna, & Sherman, 2001; Owens et al., 2018; Seltzer et al., 2012). Currently, there is no large‐scale epidemiological survey data from China. From the studies that have been carried out 29 and 30 CGG repeats alleles were the most frequent, followed by the 36 CGG repeats allele length (Chen, Lu, Che, & Ho, 1997; Pang et al., 1999). Using established FXS standards, this study used high GC content PCR to screen 10,145 women of childbearing age in China for fragile X carrier status. Sixteen female premutation carriers and two female full mutations were identified, with an overall frequency of 1/563 carriers and a premutation carrier frequency of 1/634. The objectives of the study were to promote the screening of fragile X carriers in Chinese women of childbearing age, as well as to improve the clinician's understanding of the complexities of genetic counseling on fragile X–associated diseases.

## 2 RESULTS

### 2.1 Establishment and verification of standard panel

Seventeen samples were used as part of the standard panel: four noncarrier females, two noncarrier males, two intermediate‐zone cases, two male premutation carriers, two female premutation carriers, two full mutation females, and two full mutation males, and one negative control (an *FMR1* gene‐deficient male). Each laboratory was able to accurately measure and classify the CGG repeats for these 17 standards. The mean and range of results for 12 of the DNA standards (excluding the 4 full mutations and 1 negative control) from each group are shown in Table 1. The difference of CGG repeats measurement values in the normal CGG repeat range is ±1, the range for 45–100 CGG repeats is ±5, and the range for CGG repeats over 100 is ±10. Each group performed standard measurement and repeated DNA freeze–thaw cycles three times. The CGG repeat number of the 17 standard samples was consistently and accurately classified in all laboratories.

**TABLE 1:** Summary of allele sizes and ranges for normal and premutation alleles on standard panel as measured in all laboratories

Sample ID	Gender	Allele 1			Allele 2		
		Mean	Range	SEM	Mean	Range	SEM
P1	Male	46	46–47	0.25			
P2	Male	54	54–56	0.4			
P3	Female	30	30–31	0.2	68	65–69	0.748
P4	Female	29	29–32	0.583	152	145–156	1.913
P5	Male	56	56–58	0.51			
P6	Male (mosaic)	100	94–102	1.327			
		136	128–141	2.112			
N1	Female	29	29–30	0.25	29	29–30	0.25
N2	Female	31	31	0	36	36	0
N3	Female	29	29	0	30	29–30	0.25
N4	Female	29	28–30	0.408	34	33–34	0.289
N5	Male	30	29–30	0.289			
N6	Male	36	35–36	0.25			

Abbreviation: SEM, Standard error of mean.

### 2.2 Distribution of CGG repeats in women of childbearing age

**FIGURE 1:**
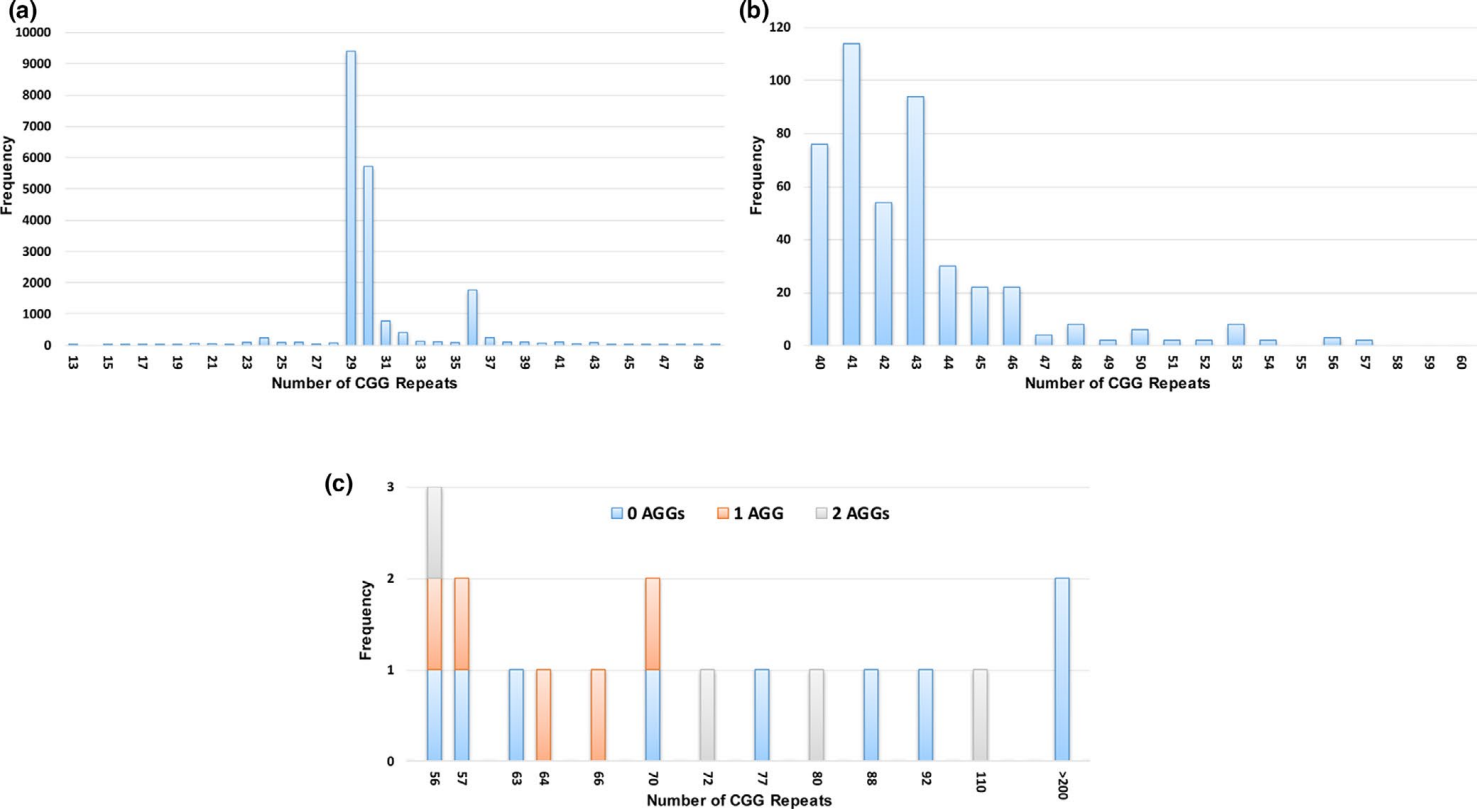
CGG repeat size allele distribution. Histograms show the frequency of alleles seen among 10,145 women. (a) *FMR1* alleles in the normal range. (b) *FMR1* alleles between 40 and 60 repeats. (c) *FMR1* alleles including number of AGG interruptions for alleles in the premutation and full mutation size range

This study analyzed CGG repeat measurements on 10,145 women of childbearing age in China (a total of 20,290 alleles). The (CGG)_29_, (CGG)_30_, and (CGG)_36_ were the most common alleles seen, accounting for 46.4%, 28.2%, and 8.8% of alleles, respectively (Figure 1a). A figure representative of PCR and Southern blot analysis results was shown in Figure S1. This was consistent with previous reports on the Chinese Han population (Huang et al., 2015).

Table 2 shows the frequency of intermediate and premutation alleles as measured in Asian and Asian American populations. For the two studies performed in the United States, the proportion of alleles from self‐reported Asians are included. Notably, for the populations studied from Taiwan, Hong Kong, or China, in general, a consistently lower prevalence of premutation alleles compared with other ethnic groups was observed. In this study, 78 intermediate‐zone alleles were identified, giving an incidence of 1/130 (95% CI: 1/104‐1/162) in the population. The identified intermediate alleles included (CGG)_45_ and (CGG)_46_ in 22 cases, (CGG)_48_ and (CGG)_53_ were seen in 8 cases, allele size of (CGG)_50_ in 6 cases, (CGG)_47_ in 4 cases, and (CGG)_49,_ (CGG)_51_, (CGG)_52_, and (CGG)_54_ were each observed in 2 cases (Figure 1b). There were 16 carriers of premutation alleles and two of full mutation alleles, giving an overall frequency of 1/563 (95% CI: 1/355‐1/894) for female carriers and premutation carrier frequency of 1/634 (95% CI: 1/388‐1/1,035). The frequency of the full mutation among women was 1/5,072 (95% CI: 1/1,269‐1/20,408). Figure 1c shows the CGG repeat numbers with the number of AGG interruptions for each of the expanded alleles.

**TABLE 2:** Reported prevalence data of FMR1 expanded alleles among Asian and Asian American populations

Year	Population	Site of recruitment	Number tested	Prevalence
2003 (Huang et al., 2003)	Pregnant women	Taiwan	1,002	Intermediate: 1/46 Premutation: 0/1002
2005 (Tzeng et al., 2005)	Newborn males	Taiwan	10,046	Intermediate: 1/143 Premutation: 1/1674
2012 (Tassone et al., 2012)	Newborn males and females	United States	428 males 368 females	Males: Intermediate: 0/428 Premutation: 1/428 Females: Intermediate: 1/74 Premutation: 1/123
2015 (Huang et al., 2015)	Unaffected males and females	China	534 males 579 females	Males: Premutation:0/534 Females: Premutation: 1/579
2017 (Cheng et al., 2017)	Pregnant women	Hong Kong	2,650	Intermediate: 1/88 Premutation: 1/1325
2018 (Owens et al., 2018)	Women referred for carrier testing	United States	7,961	Intermediate: 1/93 Premutation: 1/419
This study	Women of childbearing age	China	10,145	Intermediate: 1/130 Premutation: 1/634

## 3 DISCUSSION

In recent years, the application of noninvasive prenatal testing (NIPT) has changed the system for prenatal screening and diagnosis. NIPT, as part of the screening program for Aneuploidy and mutation detections, has been adopted in routine clinical practice rapidly and globally (Cai, Zheng, & He, 2018; Johnson & Eason, 2018). In addition, with the implementation of the two‐child policy in China, pregnant women and families are paying more attention to genetic tests to make optimal choices regarding childbearing (Li et al., 2013). In China, FXS screening will eventually become widespread, potentially facilitated by the availability of commercial FXS detection approaches. Importantly, the establishment of an FXS standard panel will ensure quality control and standardize management of commercial test kits before they enter the market. The standard panel used in this work includes intermediate repeat sizes, and it also includes two male premutation cases, two female premutation carriers, two male full mutation cases, and two female full mutation cases. The standard panel will ensure that various kits yield the same final diagnostic results. The transformation and manipulation process of EBV cell lines is complicated; therefore, DNA extractions from cell lines are selected as the standard. It is possible that cell line culturing may cause CGG size changes, so each batch of the standards will be calibrated by five Chinese institutions. Repeated testing of the standards and the recurrent freeze thaw of DNA showed consistent results, indicating that the standard panel results are reproducible and remain stable through three freeze–thaw cycles. In addition, the difference in CGG repeat measurement values for the normal CGG repeat alleles was only ±1 within all five institutions, the variance was ±5 for alleles between 45 and 100 CGG repeats, and ±10 for alleles with over 100 CGG repeats. This indicates that various detection methods can accurately classify our standard products, and the variation in CGG repeat number range is not large.

The incidence of fragile X carriers varies among different populations in the world. The rate of female premutation carriers in the European and American population is high, but there are few surveys of East Asian populations. In Taiwan, 1,002 women of childbearing age were screened for FXS and no premutation carriers were found (Tzeng et al., 2017). A study of more than 10,000 newborn males in Taiwan identified 6 premutation carriers, for a frequency of 1/1674 (Tzeng et al., 2005). The frequency of fragile X carriers was found to be 1/883 and the frequency of premutation alleles was 1/2650 among 2,650 pregnant women in Hong Kong, China (Cheng et al., 2017). In a recent large‐scale fragile X carrier screening report in the United States, the frequency of premutation carriers among those that self‐reported as Asian reached 1/419 (20/7961) in a large, ethnically diverse population referred for carrier testing (Owens et al., 2018). We previously reported the CGG repeat pattern and the *FMR1* haplotype in 1,113 Chine Han subjects and found one female carrier of a premutation allele (Huang et al., 2015). Another screening study in China demonstrated a high prevalence of 1/320 in women with abortion history, and reported 6 premutation and 2 full mutation carriers in 5,037 pregnant women (Ma et al., 2019). In this study, 10,145 Chinese women of childbearing age were screened for fragile X carrier status, and 16 female premutation carriers and 2 female full mutation carriers were found, with an overall carrier frequency of 1/563 and premutation carrier frequency of 1/634.

Woman premutation carriers in China that tested positive were most concerned about the risk of having a child with the full mutation and intellectual disability during the genetic counseling session. The majority of existing tests only detect the size of CGG repeats. Although higher CGG repeat numbers indicate a higher risk of expansion (Nolin et al., 2003), AGG interspersion patterns are also an important factor in predicting CGG repeat instability (Yrigollen et al., 2012). It has been reported that 95% of the normal *FMR1* alleles have 1–2 AGG interruptions located at the 10th to 11th and 20th to 21st regions of the 5′ end of the CGG repeat. Clinical data show that stability of the CGG repeat increases with increasing number of AGG interruptions, making them less likely to expand. However, the vast majority of premutations and full mutations lack AGG interruptions (Falik‐Zaccai et al., 1997; Nolin et al., 2013). Yrigollen et al. found that when they compared mothers with 2 AGG interruptions and an *FMR1* repeat size of ~75 repeats with mothers who had an allele with 0 AGG, their risk for having a full mutation child was decreased by more than 60%; however, once alleles were above ~90 CGG repeats in length, the presence of 1 or 2 AGG interruptions did not decrease the risk of having an offspring with a full mutation (Yrigollen et al., 2012). Therefore, the characterization of AGG interruptions is more important for premutation alleles with a smaller number of CGG repeats. Indeed, in the current dataset, the two full mutation alleles that were identified had zero AGG interruptions, as did 7 of the 16 premutation alleles that were seen, indicating the lack of AGG interruptions among unstable alleles. Risk assessment for having a full mutation child can be refined using the number of CGG repeats and number of AGG interruptions in the premutation allele. For women at higher risk, they can use their risk information to guide decision for obtaining prenatal diagnosis after natural conception or other reproductive methods such as preimplantation diagnosis. For women at low risk, doctors can recommend strategies for prenatal diagnosis after natural conception.

Currently individuals with FXS are commonly prescribed medications using a symptom‐based approach. Targeted drug development for FXS is still in the research phase, and several clinical trials are underway. Although the prevalence of the premutation allele in women from the general population is high, they are difficult to identify in absence of clinical symptoms or family history. Furthermore, in the absence of prenatal testing, they can give birth to children with intellectual disabilities. Although the identified carrier frequency in China is lower than other populations, it is imperative to educate doctors, healthcare workers, and the general public in China about the risk of FXS and associated disorders. Clearly, the establishment of an FXS standard panel will be helpful to the construction and standardization of fragile X gene detection and improve the screening for fragile X carriers in China.

## 4 MATERIALS AND METHODS

### 4.1 Ethical compliance

This study was approved by the Ethics Committee of Center for Medical Genetics, the School of Life Sciences, Central South University, and informed consent was obtained on all participants (approval number: 2013011201).

### 4.2 Study sample collection

Lymphoblastoid cell lines were established after EBV transformation for all subjects to ensure adequate samples were available in the future. Blood samples from 10,145 Chinese women of reproductive age were collected from May 2014 to June 2018 at the Center for Medical Genetics, Central South University, Changsha China. The age range of participants was from 20 to 45 years old and the participants did not report premature ovarian insufficiency or recurrent miscarriage.

### 4.3 Analysis of FMR1 CGG repeats

Genomic DNA was extracted from peripheral blood leukocytes using the proteinase‐K‐chloroform method. The number of *FMR1* CGG repeats was measured by GC‐rich PCR with the primers 5′AAGCCGGAGTCAGTCCGCGAGTCGAG3′ and 5′CACCAGCTCCTCCATCTTCTCTTCAG3′ using AmpliTaq Gold DNA polymerase (Applied Biosystems). For premutation and full mutation alleles, the AGG interruption pattern was determined by triplet‐primed (TP) PCR with the primers 5′CAGGAAACACGTATGAGGCTGCGC3′(CGG)_7_ and 5′AGAAAGCGCCATTGGAGCCCCGCACTT3′. Thermal cycling was as follows: denaturation at 98°C for 3 min, 10 cycles of 98°C for 20 s, 65°C for 45 s, and 72°C for 3 min, followed by 22 cycles of 98°C for 20 s, 68°C for 3.5 min, and a final extension at 68°C for 10 min. Each PCR product of the *FMR1* CGG repeat was analyzed on the ABI 3500 Genetic Analyzer (Applied Biosystems). GenBank reference sequence and version number used in this study was GRCh37.p13.

### 4.4 Southern blot analysis

When Southern blot was necessary, 5 µg of DNA from blood was digested with EcoR I/Eag I and hybridized with the digoxigenin‐labeled probe StB12.3 (11669940910; Roche) as described previously (Gold, Radu, Balanko, & Chiang, 2000).

### 4.5 Statistical analysis

Standardization of control samples was tested by combining the data from all laboratories for analysis. Each laboratory submitted up to three replicates per DNA sample. Standard error of mean was calculated for each DNA sample and was used as an estimate of variation between laboratories for each sample. Carrier frequencies and 95% confidence intervals (CI) were determined using a Poisson distribution in SAS 9.4.

## CONFLICT OF INTERESTS

The authors declare no competing interests.

## AUTHOR CONTRIBUTION

Ranhui Duan, Emily G Allen, Peng Jin, Kun Xia, and Juan Zhao wrote the manuscript. Fei Gao, Wen Huang, Jin Xue, Huaixing Kang, Yingbao Zhu, and Zhengmao Hu planned the study and performed the experiments. Yanjun You and Jie Huang analyzed the data.

## Supporting information

Fig S1
