# Supplementary figures and images for: Development of Chinese genetic reference panel for Fragile X Syndrome and its application to the screen of 10,000 Chinese pregnant women and women planning pregnancy
Source: Mol Genet Genomic Med. 2020 Apr 12;8(6):e1236. doi: 10.1002/mgg3.1236 (PMC7284044; doi:10.1002/mgg3.1236)

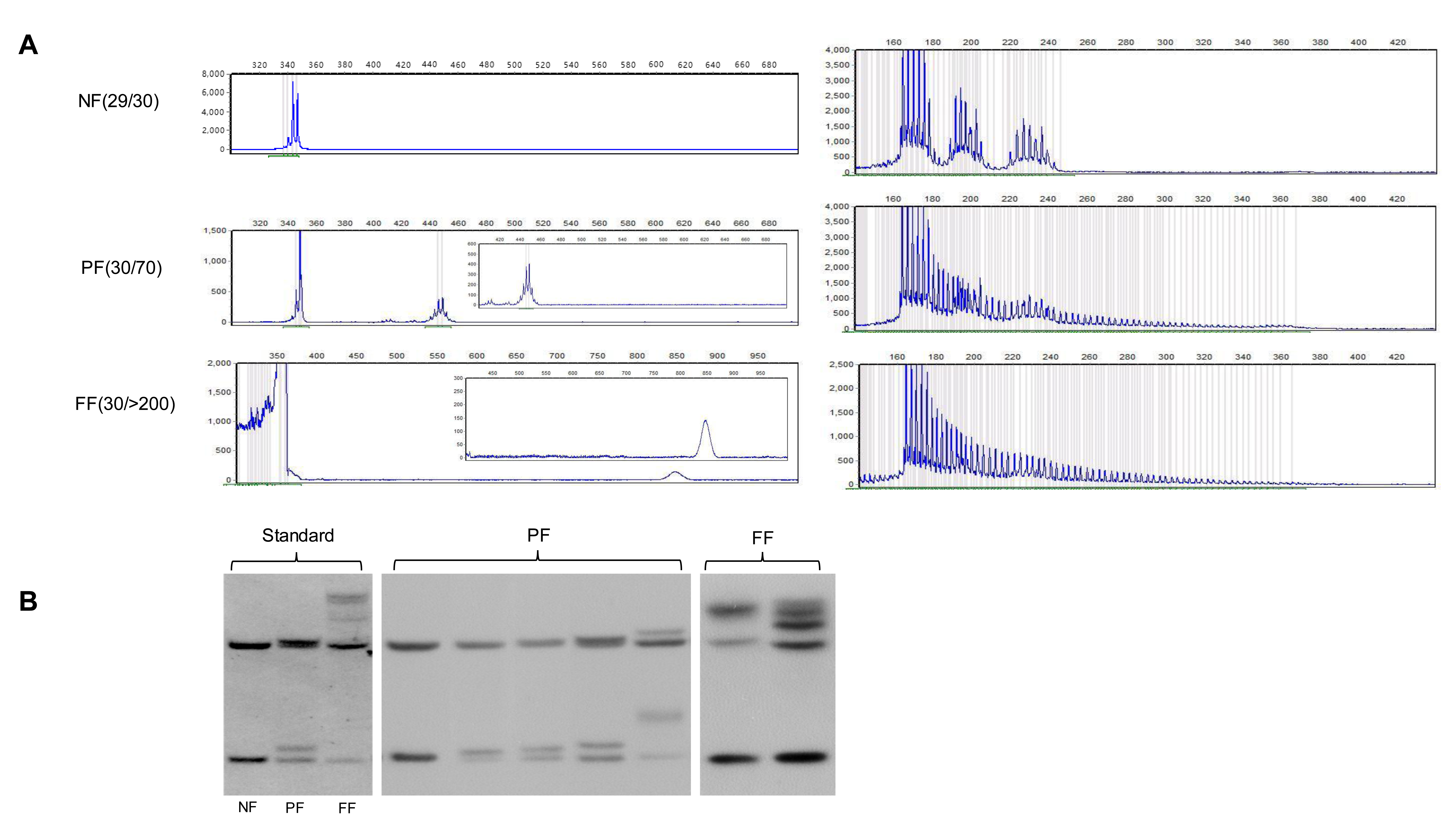

Supplement: Supplementary file 1 — Fig S1 [file MGG3-8-e1236-s001.tif]
